# Parliament and the Professions

**Published:** 1920-05-08

**Authors:** 


### Parliament and the Professions.

### Midwives.

THE following reply was made by the Minister of Health:

According to the last report of the Central Midwives Board, the number of women entitled to practise as midwives on March 31, 1919, was 44,166, but the number who gave notice of their intention to practise in 1918 was only 11,298.

No useful estimate can be made of the total number of midwives required. The shortage which exists in some districts is due to the fact that the number of cases within reach is too small to enable a midwife practising independently to make a living.

The Ministry have continued the policy of the Local Government Board of urging local authorities and nursing associations to subsidise midwifery in the more scattered districts, and of paying grants in respect of such subsidies. By this means the proportion of the rural population served by trained midwives has increased since 1917 from 51 per cent. to 65 per cent., and steady progress is being made. Nearly all the county councils and county nursing associations have framed schemes for extending the midwifery service of their counties.

A number of local authorities in urban areas have also, with the assistance of the Ministry, subsidised the provision of trained midwives in parts of their districts in need of this service.

A grant in aid of the training of women as midwives has been authorised and is being administered by the President of the Board of Education.

